# The role of intermediaries in connecting community-dwelling adults to local physical activity and exercise: A scoping review protocol

**DOI:** 10.12688/hrbopenres.13523.2

**Published:** 2022-07-07

**Authors:** Megan O'Grady, Emer Barrett, Julie Broderick, Deirdre Connolly

**Affiliations:** 1 Discipline of Physiotherapy, School of Medicine, Trinity College Dublin, the University of Dublin, Dublin, Ireland; 2 Discipline of Occupational Therapy, School of Medicine, Trinity College Dublin, the University of Dublin, Dublin, Ireland

**Keywords:** exercise, physical activity, health promotion, health services research, referral and consultation, scoping review, social prescribing, link workers

## Abstract

**Introduction**: Physical inactivity is a major global issue affecting health. Promoting, supporting and encouraging physical activity amongst community-dwelling adults is essential. An intermediary is a clinical or non-clinical professional based in primary care, community or voluntary settings. They support individuals referred to them to connect with appropriate community services with the goal of improving health and wellbeing. This may be a promising method to establish a connection to local physical activity and exercise; however the process has been poorly described to date.

**Objective**: The objectives of this scoping review will be to identify and summarise the literature describing the process of connecting community-dwelling adults to an intermediary, the characteristics of these adults, the processes (role, practice and procedure) of an intermediary in connecting these adults to local physical activity and exercise opportunities, and to map these processes of connection to outcomes.

**Methods**: This scoping review will be conducted in accordance with the scoping review methodology of the Joanna Briggs Institute. A comprehensive search strategy will identify relevant studies in Embase, Medline, Web of Science and CINAHL, along with a structured grey literature search. Studies which describe an intermediary connecting community-dwelling adults (aged ≥18 years) to local physical activity and exercise will be included. Data will be charted and narratively summarised. Intermediary processes will be mapped to outcomes related to physical activity, and the PAGER (patterns, advances, gaps, evidence for practice and research recommendations) framework will be used to identify evidence gaps and research recommendations.

**Conclusions: **This scoping review will be the first to describe the process of an intermediary connecting community dwelling adults to local physical activity and exercise. This review will identify, map and summarise the existing research on the processes and outcomes. The results will also identify any evidence gaps and will guide future research.

## Introduction

According to the World Health Organisation, over a quarter of adults are not meeting physical activity recommendations and levels of physical inactivity are increasing globally ( World Health Organization, 2019). Physical inactivity is a major modifiable risk factor for non-communicable morbidity, early mortality and results in significant direct and indirect healthcare costs ( Ding *et al.*, 2016; Lee *et al.*, 2012). Ireland has implemented several policies, strategies and monitoring systems that directly and indirectly promote increased participation in physical activity ( Bike to work, 2020; Department of Health, 2013; Department of Health, 2016; Department of Transport, 2018; Gelius *et al.*, 2021; Healthy Ireland, 2019b; Smarter Travel, 2009; Sport Ireland, 2020a; Woods *et al.*, 2010). Despite this, national data indicates that the proportion of Irish adults meeting the national physical activity guidelines remains low, with estimates ranging from 34–46% only ( Healthy Ireland, 2019a; Sport Ireland, 2020b). This declines steadily across the life course to only 18% of those aged 75 or older, falling far below the global average ( Healthy Ireland, 2019a; World Health Organization, 2019).

The health service has an essential role to “promote, educate, support and encourage physical activity generally” ( Department of Health, 2021, p. 7). At present for persons aged 15 years or older, the average number of general practitioner (GP) visits is four per year ( Collins & Homeniuk, 2021; Healthy Ireland, 2019a). This can be as high as seven per year in a chronic disease population ( Health Service Executive, 2016). Additionally, the Irish healthcare system is undergoing major reform, with significant developments underway to expand primary and community care services ( Department of Health, 2018). Therefore, primary care professionals are and will continue to be at the forefront of supporting individuals living in the community to make healthy lifestyle choices, and to support them in increasing their physical activity.

Internationally, methods of connecting community-dwelling adults from primary care services to physical activity include exercise referral schemes, brief interventions or referral to a connector or intermediary ( Albert *et al.*, 2020; Campbell *et al.*, 2015; Craike *et al.*, 2020; Cunningham *et al.*, 2021b; Leenaars *et al.*, 2018; Woods *et al.* (2016); National Institute for Health Care Excellence, 2014; Public Health England, 2021; World Health Organization, 2018). Exercise referral schemes involve referring a person from primary care services to a qualified exercise professional, to develop and deliver a tailored programme of physical activity lasting from 10–12 weeks. These schemes usually rely on partnerships between local authorities, primary care professionals and private community-based leisure service providers ( Campbell *et al.*, 2015). Recent reviews found that while the individualised support was shown to increase initial adherence to physical activity, there was marginal additional benefit on self-reported physical activity or cardiorespiratory fitness after 12 months when compared to verbal/written advice alone or signposting to local facilities ( Campbell *et al.*, 2015; National Institute for Health Care Excellence, 2014; Orrow *et al.*, 2012).

Due to the poor evidence base for exercise referral schemes, in Ireland priority has been given to health behaviour change models such as ‘Make Every Contact Count’ (MECC) instead. MECC is based on behavioural science approaches such as COM-B ('capability', 'opportunity', 'motivation' and 'behaviour') and other dual process models, which recognise the instinctive, reactive and conscious aspects of decision-making, and how internal and external cues can impact on an individual’s decisions and behaviour ( Houlihan, 2018; Michie *et al.*, 2011). The aim of MECC is to enable healthcare professionals to use their daily interactions with patients to support them in making positive health behaviour changes, including around physical activity. Interventions are opportunistic, and can range from brief advice and/or brief interventions (defined as “An intervention that aims to equip people with tools to change attitudes and explore underlying problems. It involves discussion, negotiation and encouragement” [( Health Service Executive, 2016, p22)]) with signposting to local resources, to planned, high-intensity interventions that may take place over a number of sessions ( Health Service Executive, 2016; Public Health England, 2016). 

While effective in the short term, brief interventions alone may not be sufficient to maintain improvements in physical activity, with inconclusive evidence for effectiveness on objective physical activity levels four months post-intervention ( Lamming *et al.*, 2017; Lion *et al.*, 2019; Orrow *et al.*, 2012). Lamming and colleagues (2017) reported that brief interventions may be more effective with increased follow-up and support, but evidence was mixed. Extended brief interventions of increased duration are already recommended for people requiring more intensive support for behaviour change, such as those self-managing a chronic disease ( Health Service Executive, 2016). In a more recent review of delivering brief physical activity interventions, healthcare professionals identified a common barrier of a perceived lack of local services, opportunities or places to which patients could be referred ( Hall *et al.*, 2022). If primary care professionals could therefore offer increased follow-up and support and be able to signpost their patients to appropriate services, the connection could potentially be more successful.

The final method of connection is referral to an intermediary. It has been identified as a useful way to connect people with local services and opportunities who may not benefit from brief interventions and active signposting alone ( Brandling & House, 2009; Husk *et al.*, 2020; Tierney *et al.*, 2020). There is no universal definition of an intermediary, and many titles are used to describe the role; for example, ‘care navigator’, ‘link worker’, ‘social prescriber’ or ‘sign poster’. Despite differences in title, the main attributes and work practices are similar: they are usually community-based, non-clinical staff who receive referrals for individuals with the goal of improving health and wellbeing. They connect these individuals with appropriate community and voluntary services and supports, and may follow up with the person over a number of sessions ( All Ireland Social Prescribing Network, 2021; Health Education England, 2016; Husk *et al.*, 2020; Tierney *et al.*, 2019; Tierney *et al.*, 2020).

Intermediaries can link referred individuals to a wide variety of community-based services, including physical activity. Examples of physical activity opportunities and resources from the All Ireland Social Prescribing Network website include recreational jogging groups, walking groups, virtual exercise classes and community gardening ( All Ireland Social Prescribing Network, 2021). Both primary care professionals and patients identified an intermediary as a potentially useful resource for connecting to physical activity opportunities ( Brandborg *et al.*, 2021; Carstairs *et al.*, 2020). However, in a review examining social prescribing models, less than 10% of identified studies examining exercise used an intermediary; the majority involved connecting the individual from primary care directly to an activity ( Husk *et al.*, 2020). A recent scoping review of methods of connection between primary care services and community-based physical activities found high success rates for processes that involved referral to an intermediary and/or subsequent referral to a physical activity opportunity ( Cunningham *et al.*, 2021b). However, the search was limited to evaluations undertaken in the UK, and only half of the included studies described processes which involved an intermediary. The intermediaries were poorly described, and very few studies included evaluations of effectiveness.

Current research appears to suggest that an intermediary may be a promising method of connecting community-dwelling adults to physical activity, sport, and exercise resources and opportunities in the community/local area/locality (hereafter described as local physical activity and exercise), and adults can successfully engage when referred to and supported by a link worker. However, this process has been poorly described to date. A preliminary search for existing scoping and systematic reviews of this topic was conducted using MEDLINE, the Cochrane Database of Systematic Reviews and Joanna Briggs Institute (JBI) Evidence Synthesis. Apart from the aforementioned review by Cunningham and colleagues, no other published or underway reviews were identified. The aim of this scoping review, therefore, is to identify and describe the available international evidence regarding processes and characteristics of referral to an intermediary, and the processes and outcomes of connecting referred community-dwelling adults to local physical activity and exercise via an intermediary.

## Methods

### Study design

A scoping review was deemed the most suitable methodological approach, due to the emerging and poorly delineated nature of the evidence for intermediaries and their role in physical activity promotion. A scoping review generally includes diverse sources of evidence, and can be used to clarify key concepts and knowledge gaps in a topic area ( Grant & Booth, 2009; Munn *et al.*, 2018). This review will follow the Joanna Briggs Institute guidelines for scoping reviews ( Peters *et al.*, 2020), and will be guided by the framework proposed by Arksey and O’ Malley, as well as the enhancements proposed by Levac and Daudt ( Arksey & O'Malley, 2005; Daudt *et al.*, 2013; Levac *et al.*, 2010). This protocol has been registered on the Open Science Framework ( O’Grady *et al.*, 2022).

The scoping review framework proposed by Arksey and O’Malley was employed to structure this protocol, which suggests five main steps and optional sixth step for a rigorous scoping review ( Arksey & O'Malley, 2005). These include: (1) identifying the research questions; (2) identifying relevant studies; (3) selecting studies; (4) charting the data; (5) collating, summarising, analysing and presenting the results; and (6) consulting with stakeholders to inform or validate study findings. 

### Stage 1: Identifying the research questions

The main research question, expressed using the Population-Concept-Context framework ( Peters *et al.*, 2020), is: what is the available evidence regarding the current processes (role, practice and procedure) of an intermediary, in connecting community-dwelling adults to local physical activity and exercise? In this instance, population refers to community-dwelling adults, concept refers to the work of an intermediary in establishing connections to physical activity and context refers to physical activity, sport and exercise resources and opportunities in the community/local area/locality (local physical activity and exercise).

The review objectives include the following:

i) To identify and summarise the scope of the literature describing connection to an intermediary (method and nature of referrals, number of referrals, source of referrals, reason referred, profile of the intermediaries; including background, training and sector where they are based);
ii) To identify and summarise the health characteristics and demographic information of individuals referred to an intermediary;
iii) To identify, map and summarise the available literature regarding the work of the intermediary in connecting individuals to local physical activity and exercise (process of assessment, extent and nature of community-based physical activity services and other services utilised, how these services are identified, length of follow-up, discharge from the service);
iv) To identify the available literature describing and defining outcomes of an intermediary connecting community-dwelling adults to local physical activity and exercise, and map these outcomes to processes.

The purpose of this scoping review is to gain a greater understanding of the role of an intermediary in connecting community-dwelling adults to local physical activity and exercise, to describe the core elements and common components of this process in further detail and to map the available evidence to outcomes. By fulfilling the review objectives, it is expected that evidence gaps in this topic area will emerge and inform future research recommendations. 

***Eligibility criteria***. Inclusion criteria will be limited to peer reviewed and non-peer reviewed studies that report on the process of an intermediary (concept), after receiving a referral for a community-dwelling adult (population), in connecting them to physical activity, sport and exercise resources and opportunities in the community/local area/locality (context). All full-text peer reviewed and non-peer reviewed studies which include empirical data will be considered for inclusion. Reports which do not provide primary data to answer review objectives will be excluded, such as study protocols and policy briefs. Abstracts may be included if relevant primary data is reported and study authors will be contacted to seek additional research data. Databases will be searched from inception to present. Non-English language studies will be excluded due to time and resources required for translation and interpretation. Inclusion and exclusion criteria are summarised in Table 1.

**Table 1.** Inclusion and exclusion criteria.

Inclusion criteria	Exclusion criteria
• Original empirical data: qualitative studies, quantitative studies (may include both experimental and observational trials) mixed-methods studies, and published or unpublished reports from governments and other agencies which contain primary data relevant to research objectives • Study refers to referral (of any description) of community-dwelling adults (>18 years) to an intermediary with onward referral/connection to local physical activity and exercise • Full-text peer reviewed studies • Full-text non-peer reviewed studies • English language studies	• Studies which do not contain primary data relevant to review objectives such as study protocols, policy briefs or review papers • Study participants are not community- dwelling e.g. current in-patients, living in residential care facilities • Participants are aged <18 years old • Study does not describe and/or use an intermediary to establish connection to local physical activity and exercise • Intermediary does not meet the review criteria ( *See ‘Concept’* section for further details) • Study involves physical activity delivered in a healthcare setting • Study describes other interventions (See *‘Context’* section for further details) • Full-text publication is not available after contacting authors

***Population***. The included studies must involve community-dwelling adults aged ≥18 years. Community-dwelling is defined as not living in long-term care, other residential/institutional settings or in-patient settings. Inclusion for this review will not exclude any specific clinical or diagnostic group, as this is more representative of the community-dwelling population. Because of these broad inclusion criteria, the fact that intermediaries can be based in primary care, can receive referrals from primary care, and the large volume of community-dwelling adults attending primary care services, “primary care” will be used in the search strategy to capture the population of interest. This review will include studies involving community-dwelling adults who may or may not attend primary care services, which is in line with other studies involving the same population ( Dugan *et al.*, 2001; Macmillan *et al.*, 2004; Ryan *et al.*, 2015).The terms “primary health care”, “primary care”, “general practice” and “family medicine” are often used interchangeably ( Cunningham *et al.*, 2021a) and this will be reflected in the search strategy.

***Concept***. All studies eligible for inclusion must involve referral (of any description) of community-dwelling adults to an intermediary, with an onward referral to local physical activity and exercise. We are primarily interested in the extent (breadth), range (variety) and nature (characteristics, context and design) of the process of an intermediary in connecting referred individuals to local physical activity and exercise. Almost sixty different synonyms for the role were identified on preliminary literature search carried out by the review authors, and these will be utilised in the search strategy to ensure all relevant evidence is identified. An intermediary will be defined as follows based on current literature ( All Ireland Social Prescribing Network, 2021; Health Education England, 2016; Husk *et al.*, 2020; Tierney *et al.*, 2019; Tierney *et al.*, 2020):

- Clinical or non-clinical staff, working exclusively in an intermediary role.
  This can be a dedicated intermediary post such as a link worker or social prescriber, or a healthcare professional acting as an intermediary for the purpose of the study intervention. Staff may have additional upskilling in person-centred care, health coaching and motivational interviewing;
- Based in primary care, community or voluntary sector;
- Receive self-referrals from community-dwelling adults, referrals from healthcare professionals, or referrals as part of the study intervention;
- Provide some degree of assessment and follow-up to the individual who has been referred (referee) with the aim of improving and enabling physical and mental health and wellbeing;
- Support the referee in establishing a connection to community and voluntary services offering physical activity opportunities.
  These can be pre-existing services, or developed specifically for the study intervention (see ‘ *Context’* section for further details). The intermediary can signpost or support the referee in attending, and/or follow up with them after the connection has been made, but does not deliver the physical activity intervention;
- Perform some degree of outcome measurement after connection to an activity.

An intermediary described in a study that does not meet the above criteria will not be eligible for inclusion. This working definition of an intermediary may be further refined iteratively during the review process, as we become increasingly familiar with the breadth and nature of the evidence.

***Context***. This review will include studies where the intermediary connects referred individuals to physical activity, sport and exercise resources and opportunities in the community/local area/locality (local physical activity and exercise). All types of local physical activity and exercise will be considered for inclusion, including but not limited to; walking and jogging groups (including pedometer-based walking), sports and leisure clubs, gym-based classes, exercise referral schemes (where the intermediary independently connects the referee to a programme and is not involved in delivery of the programme), adapted and chair-based exercise, outdoor activities (including gardening), yoga and Pilates. This will be reflected in the search strategy. Multimodal interventions will be included where the main focus is connection to local physical activity and exercise. Interventions where the intermediary solely/exclusively delivers general health advice or education, coaching, motivational interviewing, brief interventions (see *Introduction*) without follow-up or further signposting, exercise prescription or physical activity counselling (with no support in accessing services) will be excluded. Local physical activity and exercise that is delivered by healthcare workers or based exclusively in a healthcare setting will be excluded.

### Stage 2: Identifying relevant studies

A comprehensive search strategy aiming to identify relevant literature from a broad range of sources including electronic databases, reference lists and grey literature will be developed in conjunction with a medical librarian. As recommended by the Joanna Briggs Institute, a three-step search strategy will be utilized ( Peters *et al.*, 2020). The first step involved an initial limited search of the databases Embase and PubMed to identify studies relevant to the topic area. The second step was an analysis of the keywords and index terms of relevant studies, to create a final search strategy using all identified keywords and index terms, available as extended data (see *Extended data* ( O’Grady *et al.*, 2022)). This strategy will be undertaken across the following electronic databases, with terms modified as appropriate for each database: Embase, Medline, Web of Science and CINAHL. The third and final step will be to search the reference lists of sources that have been selected from full-text screening and grey literature searching. For the grey literature search, a search of the Canadian Agency for Drugs and Technologies in Health (CADTH) Grey Matters Tool and Google Scholar will be carried out. In addition, a search of controlled trial registers, relevant conference proceedings, academic dissertations and theses, websites of international government organizations and agencies and relevant scientific research groups and networks will also be carried out (see *Extended data* ( O’Grady *et al.*, 2022)) ( Paez, 2017). Only the first 100 hits (as sorted by relevance) from grey literature searches will be screened, as further screening will unlikely result in additional relevant literature ( Pham *et al.*, 2014; Stevinson & Lawlor, 2004). The definitive search strategy and results will be reported in detail in the published review.

### Stage 3: Selecting studies

Once the searches have been completed, results will be collated and exported to Covidence for storage, removal of duplicates, and screening against eligibility criteria. Prior to commencing the screening process, two independent reviewers will carry out pilot testing of the eligibility criteria. A random sample of 25 titles/abstracts will be selected and screened. The team will then meet to discuss any discrepancies and make modifications or refinements as needed to the eligibility criteria. Screening will commence when 75% (or greater) agreement is achieved ( Peters *et al.*, 2020). Two reviewers will independently review titles and abstracts for eligibility, meeting at the beginning, midpoint and final stages of the screening process to discuss any challenges and uncertainties ( Levac *et al.*, 2010). Disagreements will be resolved by discussion and where consensus is not achieved, a third review author will act as an arbitrator. Where inclusion or exclusion cannot be determined on the basis of title and abstract, the paper will be included for full-text screening. Included studies following review of title and abstract will be retrieved in full-text and independently reviewed by two reviewers to determine their inclusion based on the study inclusion criteria. A third author will again act as arbitrator in the case of disagreements. A final list of all included papers will be agreed amongst the research team. Reason for exclusion at all stages will be recorded and charted as part of the Preferred Reporting Items for Systematic reviews and Meta-Analyses (PRISMA) flow diagram ( Page *et al.*, 2021). 

### Stage 4: Charting the data

A data charting form will be developed *a priori* using Microsoft Excel. The tool will be designed using the JBI data extraction tool template and will include recommended charting elements, as well as other relevant data relating to the review aims and objectives ( Daudt *et al.*, 2013; Levac *et al.*, 2010; Peters *et al.*, 2020). Two reviewers will independently extract data from the first 10 included studies to pilot the data charting form, and meet to determine whether their approach to data extraction is consistent with the research question and purpose, and the suitability of the data charting form ( Daudt *et al.*, 2013; Levac *et al.*, 2010). The form may then be further refined, as familiarity with selected studies necessitates a need to capture further information ( Peters *et al.*, 2020). Any differences will be resolved by consensus discussion, with a third author available as arbitrator. 

The data extraction instrument will collect the following data relating to included studies:

1. Author(s)
2. Title
3. Year of publication
4. Evidence source details
5. Location of research (country of origin)
6. Study aims/objectives/purpose
7. Research design
8. Inclusion/exclusion criteria
9. Population of interest (health characteristics, if applicable)
10. Demographic details of participants and sample size
11. Details and features of the intermediary process and practice (method and nature of referrals, source of referrals, reason referred, number of referrals, process of assessment, length of follow-up, discharge from the service)
12. Intermediary service description, nature and approach (location, profile of the intermediaries, including background, training etc.)
13. Characteristics and details of the physical activity intervention (location, extent and nature of local physical activity and exercise services and other services utilised, how these services are identified, duration and content)
14. Presence or absence of a control/comparator group
15. Outcomes (outcomes measured, methods of measurement)
16. Other key findings that relate to the scoping review questions.

Data will be extracted by a single review author and verified by a second.

***Critical assessment for level of evidence***. As the purpose of this scoping review is to describe the current research comprehensively, papers will not be excluded based on quality criteria, therefore risk of bias assessment will not be conducted ( Arksey & O'Malley, 2005; Peters *et al.*, 2020; Peters *et al.*, 2022).

### Stage 5: Collating, summarising, analysing and presenting the results

The Preferred Reporting Items for Systematic reviews and Meta-Analyses (PRISMA) extension for Scoping Reviews (PRISMA-ScR) checklist will be used to guide the reporting of this review ( Tricco *et al.*, 2018). Results of the literature search and study screening process will be presented in a PRISMA flow diagram. Descriptive statistics will be used to summarise the general characteristics and data of the included studies. This will be presented in diagrammatic or tabular form based on the descriptive headings in the data extraction form, and accompanied by a narrative summary describing the results. This narrative summary will be grouped according to the research objectives and PCC framework ( Peters *et al.*, 2020; Peters *et al.*, 2022). A mapping exercise will be carried out to understand the relationship between the characteristics of an intermediary process and outcomes for connection to local physical activity and exercise. The PAGER framework will then be used to identify any evidence gaps. This tool was developed to provide a consistent approach to analysing, reporting and translating scoping review findings and consists of ‘patterns’, ‘advances’, ‘gaps’, ‘evidence for practice’, and ‘research recommendations’ ( Bradbury-Jones *et al.*, 2021). A patterning chart of key themes and descriptive characteristics will be produced using the data extraction form and mapping exercises, and evidence advances and gaps will be identified. Evidence for practice will identify current processes or new practices that are emerging in this field, and research recommendations will be made that are relevant and contextual to the other elements of the scoping review findings ( Bradbury-Jones *et al.*, 2021). Any deviation from the protocol will be made clear and explained in the complete scoping review report.

### Stage 6: Consulting with stakeholders to inform or validate study findings

Informal discussions took place with intermediary representatives prior to commencing the review. This engagement helped to inform the focus of the review and was instrumental in the early stages of the protocol development. We will re-engage with the stakeholders during the write-up phase to share results and discuss the implications and interpretation of this work, which will help shape the final write-up of this review. Results of this consulting step will be included in the complete scoping review report.

## Discussion

Physical inactivity is a major public health issue, and current methods aiming to increase physical activity levels have not been shown to have major long-term effects. The use of an intermediary is a promising method for establishing a successful connection to local physical activity and exercise, which in itself offers a variety of different indoor and outdoor activities, reduces barriers to participation and may improve adherence ( Carstairs *et al.*, 2020; Department of Health, 2021; Leenaars *et al.*, 2018; National Institute for Health Care Excellence, 2014). The only other scoping review to date to examine this topic found high success rates when using an intermediary to connect adults from primary care services to local physical activity and exercise, but the processes were not described in detail ( Cunningham *et al.*, 2021b).

This scoping review protocol will be the first, to the best of the author’s knowledge, to focus on the concept of an intermediary in connecting community-dwelling adults to local physical activity and exercise. It will follow a systematic approach as outlined in this protocol with a comprehensive search strategy of both peer reviewed and grey literature. This review will identify, map and summarise the existing research describing the role, process, procedure and outcomes of an intermediary in this context. The review will also identify any gaps in the research based on the review questions, and will guide future research recommendations in this topic area. 

### Dissemination

The findings of this scoping review will be published in a peer reviewed journal.

### Study status

At time of publication of this protocol, electronic database searches are underway.

## Data availability

### Underlying data

No underlying data are associated with this study.

### Extended data

Open Science Framework: The Role of Intermediaries in Connecting Community-Dwelling Adults to Local Physical Activity and Sport: A Scoping Review Protocol, http://www.doi.org/10.17605/OSF.IO/V7RBD ( O’Grady *et al.*, 2022)

This project contains the following extended data:

- Embase Search Strategy
- Grey Literature Search Strategy

Data are available under the terms of the Creative Commons Zero "No rights reserved" data waiver (CC0 1.0 Public domain dedication).

### Reporting guidelines

Open Science Framework: PRISMA-P checklist for “The Role of Intermediaries in Connecting Community-Dwelling Adults to Local Physical Activity and Sport: A Scoping Review Protocol”, http://www.doi.org/10.17605/OSF.IO/V7RBD ( O’Grady *et al.*, 2022)

Data are available under the terms of the Creative Commons Zero "No rights reserved" data waiver (CC0 1.0 Public domain dedication).
