# Supplementary material for: The role of intermediaries in connecting community-dwelling adults to local physical activity and exercise: A scoping review protocol
Source: HRB Open Res. 2022 Jul 7;5:29. Originally published 2022 Apr 8. [Version 2] doi: 10.12688/hrbopenres.13523.2 (PMC9130757; doi:10.12688/hrbopenres.13523.2)
Supplement: Peer review history

# Peer review: Reviewer response for version 1

**DOI:** 10.21956/hrbopenres.14758.r31926

**Published:** 2022-05-24

**Authors:** Caitriona Cunningham

**Affiliations:** 1 School of Public Health, Physiotherapy and Sports Science, University College Dublin, Dublin, Ireland

The proposed scoping review regarding the role of intermediaries in connecting adults with physical activity (PA) opportunities is timely and important, and the proposed methods, which are in line with Joanna Briggs Institute methodology, are robust. Overall, this protocol paper is clear and well written. The scoping review should make a valuable contribution in helping to prioritise related research and informing the implementation of physical activity pathways.

 Some points for consideration in this protocol paper and when conducting the scoping review are given below.

- Sport is mentioned in the title but not much mention of sport elsewhere. What about connecting with exercise opportunities? Would it be better to refer to physical activity and exercise instead throughout? 
- Recommend to expand the 'intermediary' definition/explanation in the abstract - it is explained in the methods of the paper.
- Context/Population - Need to clarify if referring to situations/adults where some link/engagement with the healthcare system is required for the paper to meet the inclusion criteria of scoping review.
- GP visits - 4 per year. Is this across all adult populations? Any explanator or even to say higher number likely in some cohorts, e.g. older/chronic disease pops? 
- Currently, Stage 1 objective ii) process/pathway merged with the profile of the adult population - Recommend having two separate objectives: one for population and a separate review objective to identify process/pathway for accessing intermediary and directing onwards to physical activity opportunity. Also, review and revise the stated objectives in the abstract to ensure they align well with the expanded review objectives in the Stage 1 section of the methods.
- Similarly, worth reviewing points on the data extraction tool (e.g. no. 11 - seems to be a mix of intermediary profile and actual process, should be two separate data fields).
- Some acknowledgment that brief intervention is unlikely to be sufficient to support those with more complex clinical needs/disabilities who need the support and supervision of a health care professional to adopt physical activity and exercise behaviours would be good to see.  
- Given many readers from Ireland are likely, recommend to include some information on HSE's proposed physical activity pathway to demonstrate awareness of the same. Add citation: Woods C., McCaffrey N., Furlong B., Fitzsimons-D’Arcy L., Murphy M., Harrison M., Glynn L., O’Riordan J., O’Neill B., Jennings S. and Peppard C. (2016) The National Exercise Referral Framework. Health and Wellbeing Division, Health Service Executive. Dublin. Ireland.
- 'HSCP' in the Irish context does not include doctors or nurses. Perhaps better to say healthcare professionals in the following sentence in the concept section: " *or social prescriber, or a health and social care professional acting as an intermediary...".*
- Keywords - Should a term to represent intermediary be given?

Is the study design appropriate for the research question?

Yes

Is the rationale for, and objectives of, the study clearly described?

Yes

Are sufficient details of the methods provided to allow replication by others?

Yes

Are the datasets clearly presented in a useable and accessible format?

Not applicable

Reviewer Expertise:

Physical Activity Promotion, Musculoskeletal Health and related Health Services

I confirm that I have read this submission and believe that I have an appropriate level of expertise to confirm that it is of an acceptable scientific standard.

# Response

**Published:** 2022-06-30

**Authors:** Megan O'Grady

**Affiliations:** Trinity College Dublin, Ireland

**Response to Reviewer 2:**

 Thank you so much for taking the time to review our manuscript, and for your constructive comments. Responses and subsequent changes to the manuscript are detailed below.

**Comment: **Sport is mentioned in the title but not much mention of sport elsewhere. What about connecting with exercise opportunities? Would it be better to refer to physical activity and exercise instead throughout?

**Response: **We agree that sport is not referenced often in the manuscript, and agree with the reviewer’s suggestion to use the term ‘exercise’ instead.

**Action: **References to ‘sport’ throughout the manuscript have been changed to ‘exercise’ where required/appropriate. In addition, references to ‘local physical activity’ throughout the manuscript have been changed to ‘local physical activity and exercise’ for consistency. ‘Local physical activity and exercise’ is defined as physical activity, sport, and exercise resources and opportunities in the community/local area/locality.  

**Comment: **Recommend to expand the 'intermediary' definition/explanation in the abstract - it is explained in the methods of the paper.

**Response: **We agree with this suggestion.

**Action: **The explanation of an intermediary has been expanded in the abstract introduction – “An intermediary is a clinical or non-clinical professional based in primary care, community or voluntary settings. They support individuals referred to them to connect with appropriate community services with the goal of improving health and wellbeing. This may be a promising method to establish a connection to local physical activity and exercise… ”.

**Comment: **Context/Population - Need to clarify if referring to situations/adults where some link/engagement with the healthcare system is required for the paper to meet the inclusion criteria of scoping review.

**Response: **We agree with the reviewer that this section could be clearer. We plan to include studies involving community-dwelling adults, who have been referred from a healthcare system or self-refer to intermediaries.

**Action: **The population section has been updated to include a definition of “community-dwelling” adults – “Community-dwelling is defined as not living in long-term care, other residential/institutional settings or in-patient settings”. The rationale for using “primary care” as a search term is explained, and has been updated to reflect that intermediaries receive referrals from primary care. A clarification has been added that we intend to include studies involving community-dwelling adults who may or may not attend primary care services, in keeping with other studies that involved community-dwelling adults. Additionally, in the abstract introduction, the line “… especially in primary care services where the majority of healthcare contacts take place” in relation to physical activity promotion amongst community-dwelling adults has been removed to avoid confusion.

**Comment: **GP visits - 4 per year. Is this across all adult populations? Any explanator or even to say higher number likely in some cohorts, e.g. older/chronic disease pops?

**Response: **Four GP visits per year is the average for the Irish population aged 15 years or older, and includes those who have not visited a GP. There is evidence that those with chronic disease visit their GP up to seven times a year.

**Action: **The sentence referring to GP visits in the 'Introduction' has been updated to reflect this – “At present for persons aged 15 years or older, the average number of general practitioner (GP) visits is four per year ( Collins & Homeniuk, 2021; Healthy Ireland, 2019a). This can be as high as seven per year in a chronic disease population ( Health Service Executive, 2016).”

**Comment: **Currently, Stage 1 objective ii) process/pathway merged with the profile of the adult population - Recommend having two separate objectives: one for population and a separate review objective to identify process/pathway for accessing intermediary and directing onwards to physical activity opportunity. Also, review and revise the stated objectives in the abstract to ensure they align well with the expanded review objectives in the Stage 1 section of the methods.

**Response: **We agree with the reviewer that separating the population and the process objectives is more appropriate.

**Action: **Study objective (i) has been amended to, “To identify and summarise the scope of the literature describing connection to an intermediary (method and nature of referrals, source of referrals, reason referred, number of referrals, profile of the intermediaries; including background, training and sector where they are based)”; and study objective (ii) has been amended to, “To identify and summarise the health characteristics and demographic information of individuals referred to an intermediary”. The abstract has been updated under the ‘Objective’ section to include study objective (ii).

**Comment: **Similarly, worth reviewing points on the data extraction tool (e.g. no. 11 - seems to be a mix of intermediary profile and actual process, should be two separate data fields).

**Response: **We have reviewed the data fields on the proposed data extraction instrument and updated them where necessary to reflect the study objectives.

**Action: **Data fields 11 and 12 have been updated as follows: “11. Details and features of the intermediary process and practice (method and nature of referrals, source of referrals, reason referred, number of referrals, process of assessment, length of follow-up, discharge from the service)” and “12. Intermediary service description, nature and approach (location, profile of the intermediaries, including background, training etc.)”

**Comment: **Some acknowledgment that brief intervention is unlikely to be sufficient to support those with more complex clinical needs/disabilities who need the support and supervision of a health care professional to adopt physical activity and exercise behaviours would be good to see.

**Response: **People living with chronic disease or who need more intensive healthcare support are recommended to have extended brief interventions in order to achieve health behaviour change. This is reflected in the Making Every Contact Count Framework and Implementation Plan for Health Professionals in the Irish Health Service.

**Action: **A line has been added to the ‘Introduction’ to reflect this – “Extended brief interventions of increased duration are already recommended for people requiring more intensive support for behaviour change, such as those self-managing a chronic disease ( Health Service Executive, 2016).

**Comment: **Given many readers from Ireland are likely, recommend to include some information on HSE's proposed physical activity pathway to demonstrate awareness of the same. Add citation: Woods C., McCaffrey N., Furlong B., Fitzsimons-D’Arcy L., Murphy M., Harrison M., Glynn L., O’Riordan J., O’Neill B., Jennings S. and Peppard C. (2016) The National Exercise Referral Framework. Health and Wellbeing Division, Health Service Executive. Dublin. Ireland.

**Response: **The reference has been included as a source of information for those readers requiring further detail on the National Exercise Referral Framework in Ireland.

**Action: **The reference has been added to the paragraph in the 'Introduction' explaining exercise referral schemes.

**Comment: **'HSCP' in the Irish context does not include doctors or nurses. Perhaps better to say healthcare professionals in the following sentence in the concept section: "or social prescriber, or a health and social care professional acting as an intermediary...".

**Response: **We agree with the reviewer’s suggestion.

**Action: **References to ‘health and social care professional/s’ in the ‘Concept’ section have been updated to ‘healthcare professional/s’.

**Comment: **Keywords - Should a term to represent intermediary be given?

**Response: **‘Social prescribing’ was chosen as a keyword to represent intermediaries, due to the variation in nomenclature internationally. In addition, ‘link workers’ has also been used in the literature.  

**Action: **‘Link workers’ has been added as a keyword.

# Peer review: Reviewer response for version 1

**DOI:** 10.21956/hrbopenres.14758.r31853

**Published:** 2022-05-06

**Authors:** Joey Murphy

**Affiliations:** 1 Centre for Exercise, Nutrition and Health Sciences, School for Policy Studies, University of Bristol, Bristol, UK

This manuscript presents a scoping review protocol that aims to identify and summarise the literature describing the process of connecting community-dwelling adults to an intermediary, the processes (role, practice, and procedure) of an intermediary in connecting these adults to local physical activity and sports opportunities, and to map these processes of connection to outcomes.

 First, I would like to thank the authors and editorial team for this opportunity to review this work. Overall, the protocol is well-written with a clear justification and proposed methods presented. I have minor feedback that I feel will strengthen this work.

**Introduction**

 The introduction is well-written with a clear justification for this scoping review provided. The authors clearly describe the current context in Ireland which will help translate this work for international readers. I have no revisions for this section.

**Methods**

 Again, the methods present a clear overview of the steps that will be taken in order to complete this scoping review in a credible and rigorous way. It is great to see the authors using a combination of the JBI guidelines, Arksey and O'Malley framework, and enhancements from Levec and Daudt to guide this review. 

I have three suggestions for this section:

1. I see from the end of the document that this scoping review is registered through the Open Science Framework. I think it would be useful to state this in-text so readers are aware. 
2. When explaining the eligibility criteria you state: "Inclusion criteria will be limited to studies that report on...". I think it would be good to highlight that you mean both peer and non-peer reviewed studies. This would align with the criteria listed in Table 1. 
3. You do a good job justifying the limitations of previous work and why this research is needed during the introduction. However, considering one difference between this review and Cunningham's is looking at community-dwelling versus primary care populations specifically, I think it would be a good idea to define "community-dwelling". The current "Population" section sounds like you are focusing on primary care, when in fact I think you are looking at a wider group. I think highlighting that community-dwelling populations are much wider and defining this would improve the section. 

Is the study design appropriate for the research question?

Yes

Is the rationale for, and objectives of, the study clearly described?

Yes

Are sufficient details of the methods provided to allow replication by others?

Yes

Are the datasets clearly presented in a useable and accessible format?

Not applicable

Reviewer Expertise:

Physical activity, public health, pragmatic evaluation, implementation, systems thinking, knowledge translation

I confirm that I have read this submission and believe that I have an appropriate level of expertise to confirm that it is of an acceptable scientific standard.

# Response

**Published:** 2022-06-30

**Authors:** Megan O'Grady

**Affiliations:** Trinity College Dublin, Ireland

**Response to Reviewer 1:**

 Thank you so much for taking the time to review our manuscript, and for your constructive comments. Responses and subsequent changes to the manuscript are detailed below.

**Comment:** The introduction is well-written with a clear justification for this scoping review provided. The authors clearly describe the current context in Ireland which will help translate this work for international readers. I have no revisions for this section.

**Response:** Thank you for your comments.

**Action:** None required.

**Comment:** I see from the end of the document that this scoping review is registered through the Open Science Framework. I think it would be useful to state this in-text so readers are aware.

**Response: **This is useful information for the reader.

**Action: **A line has been added to the ‘Study design’ section of the Methods to indicate the protocol is registered through the Open Science Framework.

**Comment: **When explaining the eligibility criteria you state: "Inclusion criteria will be limited to studies that report on...". I think it would be good to highlight that you mean both peer and nonpeer reviewed studies. This would align with the criteria listed in Table 1.

**Response: **This will align the text with the inclusion/exclusion criteria listed in Table 1.

**Action: **Reference to “studies” in the eligibility criteria section changed to “peer reviewed and non-peer reviewed studies”.

**Comment: **You do a good job justifying the limitations of previous work and why this research is needed during the introduction. However, considering one difference between this review and Cunningham's is looking at community-dwelling versus primary care populations specifically, I think it would be a good idea to define "community-dwelling". The current "Population" section sounds like you are focusing on primary care, when in fact I think you are looking at a wider group. I think highlighting that community-dwelling populations are much wider and defining this would improve the section.

**Response: **We agree with the reviewer’s comment that this section needs a clearer definition of “community-dwelling”. We are intending to include studies involving a wider group, not just those who attend primary care services.  

**Action: **The population section has been updated to include a definition of “community-dwelling” adults – “Community-dwelling is defined as not living in long-term care, other residential/institutional settings or in-patient settings”. The rationale for using “primary care” as a search term is explained and has been updated to reflect that intermediaries receive referrals from primary care. A clarification has been added that we intend to include studies involving community-dwelling adults who may or may not attend primary care services, in keeping with other studies that involved community-dwelling adults.
